# Vasoactive Intestinal Peptide and Its Receptors in Human Ovarian Cortical Follicles

**DOI:** 10.1371/journal.pone.0037015

**Published:** 2012-05-18

**Authors:** Rinat Gabbay-Benziv, Asangla Ao, Benjamin Fisch, Li Zhang, Galia Oron, Gania Kessler-Icekson, Avi Ben-Haroush, Haim Krissi, Ronit Abir

**Affiliations:** 1 Infertility and IVF Unit, Beilinson Women’s Hospital, Rabin Medical Center, Petach Tikva and Sackler Faculty of Medicine, Tel Aviv University, Tel Aviv, Israel; 2 Department of Obstetrics and Gynecology, Royal Victoria Hospital, McGill University, Montreal, Quebec, Canada; 3 Department of Human Genetics, McGill University, Montreal, Quebec, Canada; 4 The Felsenstein Medical Research Center, Beilinson Hospital, Petach Tikva and Sackler Faculty of Medicine, Tel Aviv University, Tel Aviv, Israel; 5 Beilinson Women’s Hospital, Rabin Medical Center, Petach Tikva and Sackler Faculty of Medicine, Tel Aviv University, Tel Aviv, Israel; University of Kansas Medical Center, United States of America

## Abstract

**Background:**

Ovarian cryopreservation is one option for fertility preservation in patients with cancer. The danger of reseeding malignancies could be eliminated by *in vitro* maturation of primordial follicles from the frozen-thawed tissue. However, the development of this system is hindered by uncertainties regarding factors that activate primordial follicles. Neuronal growth factors such as vasoactive intestinal peptide (VIP) play important roles in early mammalian folliculogenesis. There are no data on the expression of VIP and its vasoactive intestinal peptide pituitary adenylate cyclase 1 and 2 receptors (VPAC1-R and VPAC2-R) in human preantral follicles.

**Methodology/Principal Findings:**

Tissue samples from 14 human fetal ovaries and 40 ovaries from girls/women were prepared to test for the expression of VIP, VPAC1-R, and VPAC2-R on the protein (immunohistochemisty) and mRNA (reverse transcription polymerase chain reaction) levels. Immunohistochemistry staining was mostly weak, especially in fetal samples. The VIP protein was identified in oocytes and granulosa cells (GCs) in the fetal samples from 22 gestational weeks (GW) onwards. In girls/women, VIP follicular staining (oocytes and GCs) was identified in 45% of samples. VPAC1-R protein was identified in follicles in all fetal samples from 22GW onwards and in 63% of the samples from girls/women (GC staining only in 40%). VPAC2-R protein was identified in follicles in 33% of fetal samples and 47% of the samples from girls/women. The mRNA transcripts for VIP, VPAC1-R, and VPAC2-R were identified in ovarian extracts from fetuses and women.

**Conclusions:**

VIP and its two receptors are expressed in human ovarian preantral follicles. However, their weak staining suggests they have limited roles in early follicular growth. To elucidate if VIP activates human primordial follicles, it should be added to the culture medium.

## Introduction

One option for fertility preservation in female patients with cancer is cryopreservation of ovarian tissue containing primordial follicles [1]–[3]. Studies reported that reimplantation of frozen-thawed ovarian tissue from cancer patients resulted in livebirths [3]. However, the procedure carries a risk of transmitting malignancies [2], [4], which could be eliminated by *in vitro* maturation of primordial follicles [4], [5]. The development of a successful *in vitro* maturation system is currently hindered by uncertainties regarding the factors that promote primordial follicular development.

There is increasing evidence of the importance of neuronal growth factors such as neurotrophins in ovarian function [6]–[11]. Neuronal growth factors are probably also involved in early folliculogenesis, including activation of primordial follicles. One such factor is the neuropeptide vasoactive intestinal peptide (VIP) [12]. It is a 28-amino acid peptide derived from a 170-amino acid precursor (PreProVIP), that was first isolated in 1970 from extracts of porcine duodenum [13], [14]. VIP is one of the major peptide transmitters in the central and peripheral nervous system and has a variety of biological actions including vasodilatation, relaxation of gastrointestinal smooth muscle and release of hormones from the pancreas and adenohypophysis [15]. Its activities are mediated by two classes of G-protein-coupled receptors namely vasoactive intestinal peptide pituitary adenylate cyclase 1 or 2 receptors (VPAC1-R and VPAC2-R) [16]. The VIP receptors are characterized by large, complex N-terminus domains that contain a high concentration of cysteine residues. The first and second extracellular loops also contain a high concentration of cysteine residues which participate in disulfide bonds essential in maintaining ligand-binding topology. Receptor function is coupled to adenylate cyclase or phospholipase C or D activation [17].

There is limited information on the expression of VIP or its receptors in mammalian preantral ovarian follicles. In experimental studies, VPAC1-R and VPAC2-R were detected in the ovaries of sexually mature mice [18], and VIP was detected at the onset of follicular development in bovine fetuses with an increase in expression with gestational age [15]. *In vitro* exposure of neonate rat ovaries (containing either primordial follicles or follicles during follicular assembly) to VIP increased levels of cytochrome P-450 aromatase (P-450arom) and mRNA transcripts of follicle stimulating hormone (FSH) receptor [19]. Similarly, in goats, the presence of VIP improved preantral follicular survival and development [12].

In humans, the expression of VIP and its receptors in preantral follicles has not been investigated. The aim of the present study was to determine the expression of VIP and its two receptors in human ovarian cortical follicles from fetuses, girls, and women on both the protein level, by immunohistochemistry (IMH) and the messenger RNA (mRNA) level, by reverse transcription polymerase chain reaction (RT-PCR). Indentifying the VIP system in human primordial follicles will suggest possible VIP involvement in their activation.

## Results

### IMH Detection of VIP, VPAC1-R and VPAC2-R Proteins

Table 1 summarizes the IMH findings for the expression of VIP and its receptors in ovarian samples from fetuses, girls and women. Positive red-brown staining for VIP, VPAC1-R and VPAC2-R was detected in all the sections tested. In the oocyte, findings were subclassified as nuclear staining and cytoplasmic staining. Positive oocyte staining was described as either full (staining in the whole cytoplasm) or partial (staining only in part of the cytoplasm). A follicle with at least one red-brown granulosa cell (GC) was defined as positively stained in its GCs. Negative staining was defined as an absence of red-brown staining (only blue counterstaining).

**Table 1:** Protein expression (IMH) of VIP, VPAC1-R and VPAC2-R in human ovaries.

Source	VIP	VPAC1-R	VPAC2-R
*Fetus*	*Weak*	*Weak*	*Weak*
Oocyte	+ From 22GW	+ From 22GW	+33%
Cytoplasm	+ Full from 22GW	+ Full from 22GW	+ Full
Nucleus	+ From 22GW	+ From 22GW	+
GCs	+ From 22GW	+ From 22GW	+
Stroma	+ All samples	+ All samples	+ All samples
***Girls/Women***	***Weak-Medium***	***Weak***	***Weak-Medium***
Oocyte	+45%	+63%	+47%
Cytoplasm	Full (70%)/Partial (30%)	+ Full	Full (63%)/Partial (37%)
Nucleus	+	+	+
GCs	+	+40% of stained	+ all stained
Stroma	+ All samples	+ All samples	+ All samples

Note: IMH = immunohistochemistry, GW = gestational weeks. Percentages represent the proportion of the samples with follicular staining.

Staining intensities: + = positive staining; full/partial = full or partial cytoplasmic staining.

**Figure 1:**
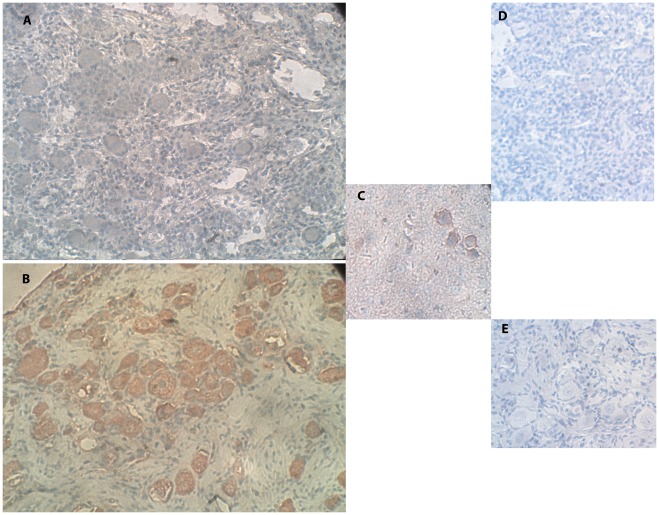
IMH photographs of VIP protein expression. (A) Section of human ovary from a 22-GW-old fetus. Note the primordial follicles, weak red-brown staining indicating VIP expression in oocytes (full cytoplasmic and nuclear staining), and weak staining in a portion of the GC and stroma cells. Original magnification X400. (B) Section of human ovary from a 6-year-old girl. Note the primordial follicles with red-brown staining indicating VIP protein expression in the oocyte (mainly cytoplasmic staining and nuclear staining), and in a portion of the GC and a portion of the stroma cells. Original magnification X400. (C) Positive control for VIP protein expression of section of mouse brain. Note the red-brown staining in the sample. Original magnification X400. (D) Negative control for the same ovarian section as in panel A. Note the primordial follicles with the overall blue staining and the lack of red-brown staining. Original magnification X400. (E) Negative control for the same ovarian section as in panel B. Note the primordial follicles with overall blue staining and the lack of red-brown staining. Original magnification X400.

The characteristic cellular localization of the VIP protein in fetuses is represented in Figure 1A. Staining was weak. Follicular staining was identified from 22 gestational weeks (GW) onwards. Full positive staining in the cytoplasm with nuclear staining was identified in all of these oocytes and in a portion of the GCs of each follicle. The characteristic cellular localization of VIP in girls/women is represented in Figure 1B. Follicular staining was identified in 45% of the samples, in which oocyte cytoplasmic staining was full in 70% and partial in 30% with nuclear staining; a portion of the GCs of all these specimens stained positively. The positive control stained positively (Figure 1C).

**Figure 2:**
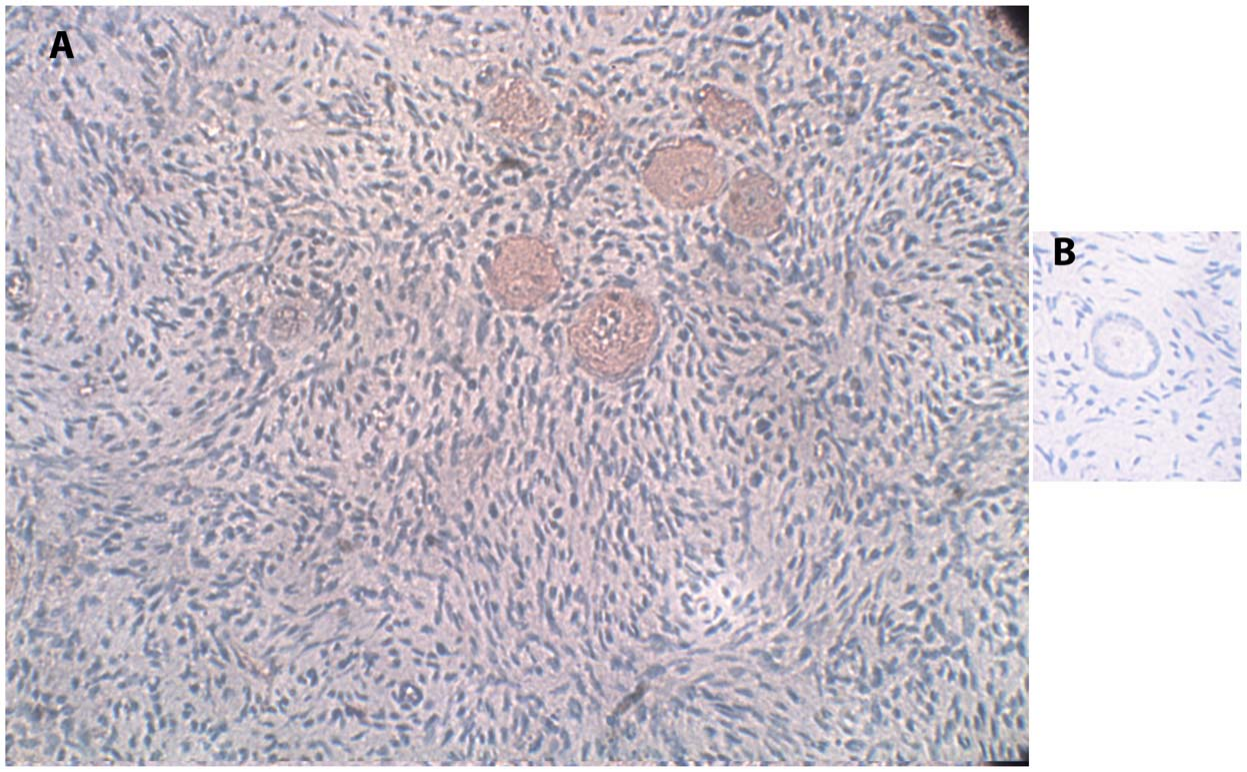
IMH photographs of VPAC1-R protein expression. (A) Section of human ovary from a 22-year-old woman. Note the primordial follicles, with red-brown staining indicating VPAC1-R expression in the oocytes (full cytoplasmic staining and nuclear staining), and in a portion of the GC and stroma cells. Original magnification X400. (B) Negative control for the same ovarian section as in panel A. Note the primordial follicle, overall blue staining, and lack of red-brown staining. Original magnification X400.

The characteristic cellular staining of the VPAC1-R protein in fetuses was very weak, and therefore, no picture is presented. Follicular staining was identified from 22 GW onwards. Full positive staining in the cytoplasm with nuclear staining was detected in all oocytes and in the GCs. The characteristic cellular localization of the staining for VPAC1-R in girls/women is represented in Figure 2A. Follicular staining was identified in 63% of the samples, in all of which oocyte cytoplasmic staining was full with nuclear staining. Staining in GCs was identified in 40% of the specimens and only in a portion of the GCs of each follicle.

**Figure 3:**
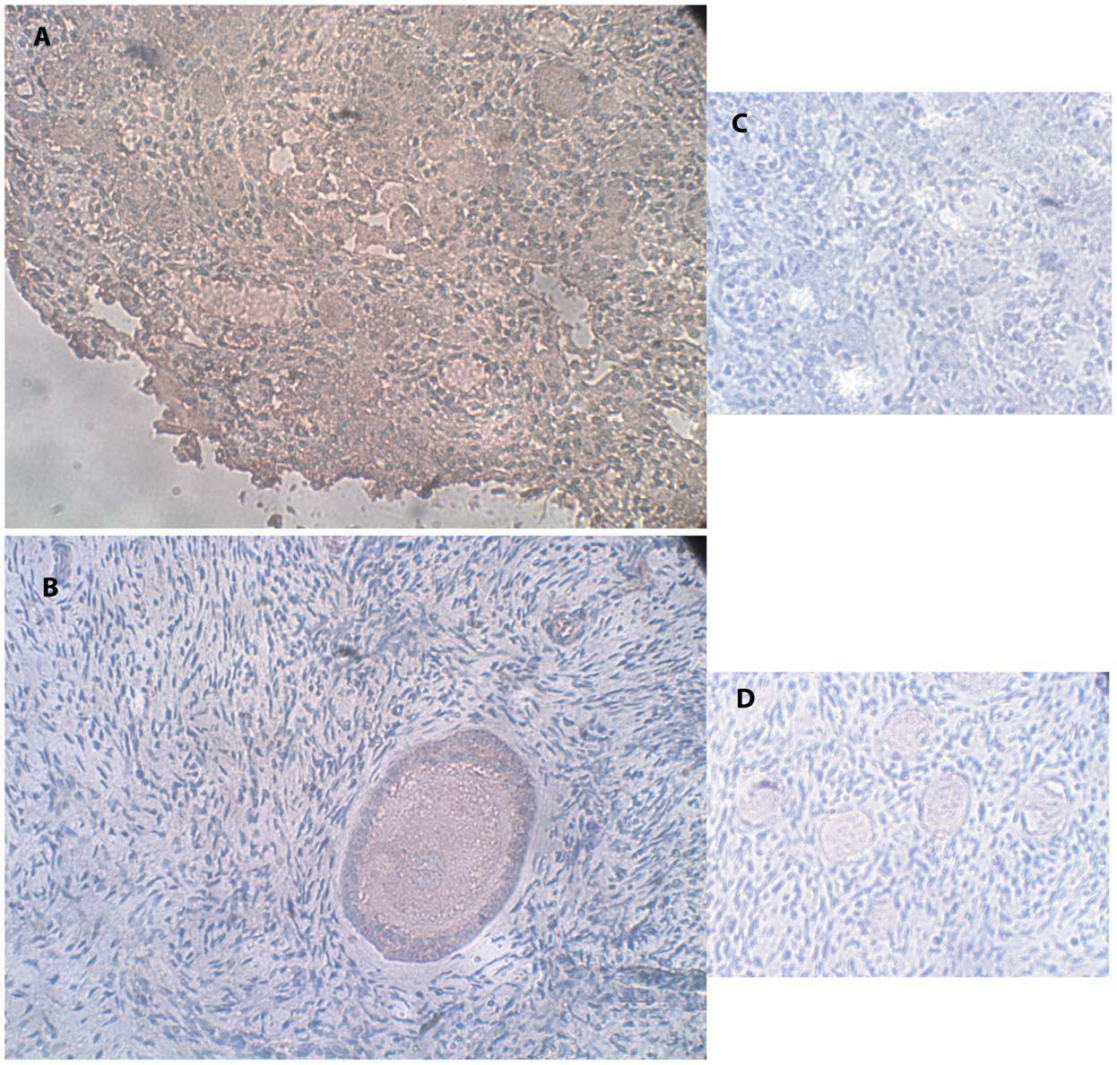
IMH photographs of VPAC2-R protein expression. (A) Section of a human ovary from the same fetus as in Figure 1 (A) and (D). Note the red-brown staining indicating VPAC2-R expression in the oocytes (full weak cytoplasmic staining with nuclear staining), in a portion of the GC and stroma cells. Original magnification X400. (B) Section of human ovary from the same woman as in Figure 2 (A) and (B). Note the secondary follicle (full cytoplasmic staining with nuclear staining), with red-brown staining in the GC and stroma cells. Original magnification X400. (C) Negative control for the same ovarian section as in panel A. Note the primordial follicles with overall blue staining and lack of red-brown staining. Original magnification X400. (D) Negative control for the same ovarian section as in panel B. Note the primordial follicles with overall blue staining and lack of red-brown staining. Original magnification X400.

The characteristic cellular localization of the VPAC2-R protein in fetuses is represented in Figures 3A. Staining was weak. Follicular staining was identified regardless of gestational age in 33% of the samples, in which oocyte cytoplasmic staining was full with nuclear staining. Positive staining was identified in a portion of the GC of each follicle. The characteristic cellular localization for VPAC2-R in girls/women is represented in Figure 3B. Follicular staining was identified in 47% of the samples, in which oocyte cytoplasmic staining was full in 63% and partial in 37% with nuclear staining in all. Positive staining was identified in a portion of the GCs of each follicle.

A portion of the stroma cells in all the samples expressed the proteins for VIP, VPAC1-R and VPAC2-R. There was no further association of the results with ovarian source (fetus, girls and women), fetal abnormalities, age, or follicular class. The negative controls for all three proteins stained only with the blue hematoxylin counterstain (Figures 1D, Figure 1E, 2B, 3C and 3D).

### Follicular Counts

In the samples from fetuses on IMH studies, 1584 follicles were counted: 1456 (91.9%) primordial and 127 (8%) primary. In the samples from girls/women, 952 follicles were counted: 767 (80.6%) primordial, 169 (17.8%) primary, 14 (1.5%) secondary and 1 antral (0.1%).

### RT-PCR Results

**Figure 4:**
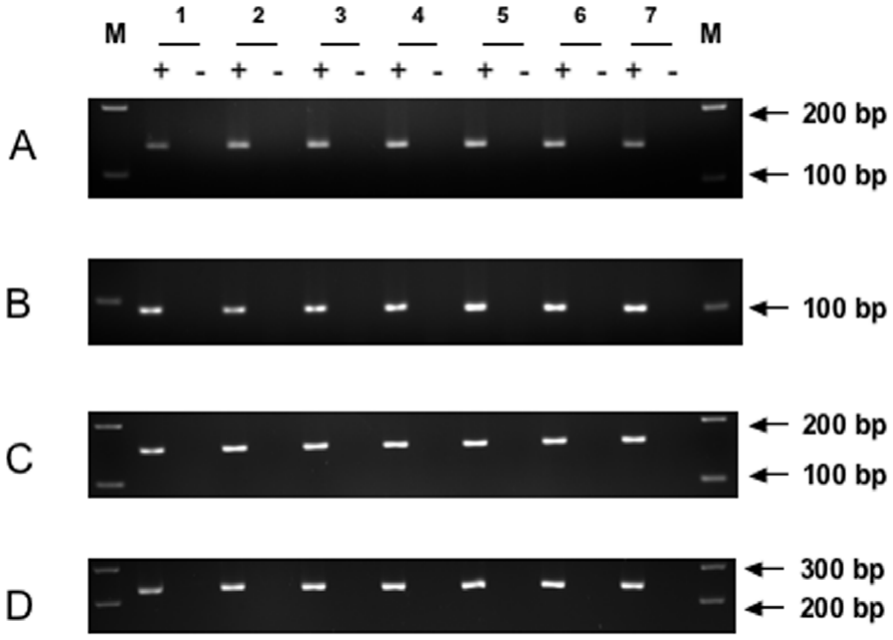
Representative RT-PCR gel illustrating expression of the VIP, VPAC1-R and VPAC2-R genes in fetal and adult ovaries. (A) VIP gene (134 base pairs), (B) VPAC1-R gene (97 base pairs), (C) VPAC2-R gene (155 base pairs), (D) DHFR gene as positive control (231base pairs). Sample 1: Ovary from a 21-year-old woman, Sample 2: Ovary from a 29-year-old woman, Sample 3: Ovary from a 39-year-old woman, Sample 4: Ovary from a 21-GW-old fetus, Sample 5: Ovary from a 23-GW-old fetus, Sample 6: Ovary from a 25-GW-old fetus, Sample 7: Ovary from a 27-GW-old fetus. M: Marker (100 base pair DNA ladder, QIAGEN), +: RT presence, -: RT absence.

RT–PCR analysis was performed on eight samples from women and eight samples from fetuses (Figure 4). All genes studied yielded the expected fragments in all samples from both sources. Positive findings were also obtained for the control dihydrofolate reductase (DHFR) gene. None of the negative controls processed without RT (RT-) yielded an amplification product.

## Discussion

Our study is the first to provide information on the presence of VIP, VPAC1-R and VPAC2-R in human cortical follicles from fetuses, girls, and women. The VIP protein was identified in oocytes and GCs in all samples from fetuses aged ≥22GW. In girls/women, the VIP protein was identified in follicles in 45% of samples (full cytoplasm staining in 70% of oocytes, partial 30%). The VPAC1-R protein was identified in follicles of all fetal samples from 22GW onwards and in 63% of the samples from girls/women (with GC staining in 40%). The VPAC2-R protein was identified in follicles in 33% of the fetal samples and 47% of the samples from girls/women (full oocyte cytoplasmic staining in 63%, partial in 37%). IMH staining was mostly weak, especially in the fetal samples. The mRNA transcripts for VIP, VPAC1-R and VPAC2-R were identified by RT-PCR in the ovarian extracts from fetuses and women.

In general, we found a good correlation in expression of the proteins for VIP and its receptors between the protein and the mRNA levels. The weaker protein staining for VIP and its two receptors in the fetal samples than in the samples from girls/women may be attributed to age-related developmental changes. This is also in line with the finding of fetal staining only from 22 GW onwards. However, protein staining was weak in the fetal samples, while the corresponding mRNA transcripts were easily identified. This finding might be attributed to a higher sensitivity of RT-PCR to low levels of mRNA transcripts relative to the sensitivity of IMH to the relevant proteins [20]. In addition, our RT-PCR assay was qualitative and not quantitative and, therefore, could not detect changes in mRNA levels. Given that the protein staining for VIP and/or its two receptors was mostly weak and not present in all follicles from girls/women, it seems likely that the VIP system plays a limited and as yet unknown role in early follicular growth in humans.

Some information is available in the literature about VIP and its two receptors in preantral follicles in other mammals [12], [15], [18], [19], [21], [22]. In a study of 22-day-old mice no mRNA transcripts for VIP were identified, but VPAC1-R and VPAC2-R were detected in the ovaries at both the protein level (Western blot) and mRNA level (RT-PCR) [18]. The two receptors were identified in residual ovarian tissue, and VPAC2-R also in GC. It is noteworthy that VPAC1-R was downregulated by human chorionic gonadotrophin [18]. In a study of bovine ovaries, VIP was detected from four and a half to six months of gestation coinciding with the onset of follicular development; its expression increased with gestational age [15]. VIP immunoreactivity was limited to the ovarian cortex up to the age of nine months of gestation, and thereafter was found in the medulla as well around blood vessels and nonvascular smooth muscle cells, and near preantral follicles. After birth, the number of VIP immunoreactive sites gradually increased with age in both the ovarian cortex and medulla [15]. The age-related changes in the bovine are comparable with our findings of a weaker expression of the proteins for VIP and its two receptors in human fetal ovaries (only from 22 GW onwards) than in ovaries from girls/women. Differences between our results in humans and those in mice [18] and cows [15] can be attributed to species differences. Moreover, unlike the bovine study [15] we did not study the ovarian human medulla, as it was removed from the cortex either during the operation itself or during tissue preparation before cryopreservation. Indeed, a recent study identified preantral follicles also in the human medulla [23], but we were unaware of these findings at the time of tissue collection.

There seem to be complex interactions between VIP and FSH at least in rodents. Therefore, VIP might be a regulator of FSH function [19], [21]. The addition of VIP to preantral follicles from adult mice that were already under FSH induction caused a dose-dependent inhibition of follicular growth, antrum formation, GC proliferation, and estradiol production [21]. In the neonatal rat ovary (containing mostly primordial follicles), VIP caused (within eight hours of treatment) a dose-dependent increase in FSH receptor mRNA accompanied by the formation of functional receptor molecules, as demonstrated by the ability of FSH to stimulate the formation cyclic adenylate monophosphate [19]. At the same time, VIP also increased aromatase activity prior to folliculogenesis [22] and the steady state levels of P-450arom mRNA [19]. In the goat, VIP improved preantral follicular survival and development after *in vitro* culture and assisted in maintaining follicular ultrastractural integrity on transmission electron microscopy study [12].

The studies in mammals [12], [15], [18], [19], [21], [22] and the present study in humans, all suggest that VIP plays a role in early follicular development. Although the precise function of VIP in primordial/preantral follicles is still unclear, it seems to be species and age-dependent (increases with age) and to involve interactions with other factors. Some of the studies showed that VIP alone stimulated the growth of preantral follicles [12], [19], [22], whereas its synergism with FSH inhibited follicular growth [21]. In future studies, to elucidate if VIP is involved in the activation of human primordial follicles, it could be added to the culture medium without any additional factor [12], [19], [21], [22]. At the same time, the limited staining of VIP and its two receptors in human preantral follicles, suggests a limited and as yet unknown function of VIP in early follicular development in humans. Therefore, further studies of the association of other growth factors with the development of human primordial follicles are needed.

## Materials and Methods

### Human Ovaries from Fetuses, Girls and Women

Ovarian samples were obtained from 14 human fetuses aged 21–33 GW, following approved pregnancy terminations (performed mostly because of fetal abnormalities) [8], [10], [11]. In addition, small ovarian cortical biopsy samples were donated by 40 girls/women aged 5–39 years or their parents. All had undergone ovarian laparoscopies for either cryopreservation of ovarian tissue before commencement of chemotherapy or for removal of ovarian cysts. The Ethics Committee of Rabin Medical Center approved the study, and every patient or minor’s parents signed an informed consent form.

The samples were cut to uniform-size and fixed immediately. The remaining sample material was frozen until RNA extraction [8], [10], [11].

### Cryopreservation of Ovarian Tissue

Tissue slices were placed in cryogenic vials (Nalge Nunc International, Roskilde, Denmark) with a solution of dimethylsulfoxide (1.5 M) (Sigma, St. Louis, USA) [8], [10], [11]. Prior to freezing, the samples were kept on ice for 30 minutes to reach equilibrium with the solution. The specimens were then frozen in a programmable freezer (Kryo 360-1/7, Planer Biomed, Sunbury on Thames, UK), and immediately placed in liquid nitrogen. The slices were cryostored until RNA extraction.

### Histological Preparation

Our histological preparation method has been described in detail elsewhere [8], [10], [11]. The fixed specimens were dehydrated in a graded series of ethanol and toluene followed by paraffin embedding and sectioning. Unstained sections were placed on OptiPlus positive-charged microscope slides (BioGenex Laboratories, San Ramon, CA, USA) for IMH.

### IMH for VIP, VPAC1-R and VPAC2-R

Our IMH methods have been described in detail elsewhere [8], [10], [11]. In brief, two sections per sample were utilized for the identification of the VIP, VPAC1-R and VPAC2-R proteins. To enhance antigen retrieval, all slides were microwaved with citrate buffer at pH 6.0 (CheMate buffer, DAKOCytomation, Glostrup, Denmark), and to block endogenous peroxidase activity, the slides were quenched in 3% hydrogen peroxide (H_2_O_2_, Vitamed, Binyamina, Israel). A mouse brain sample served as a positive control for VIP (according to instructions from Santa Cruz Biotechnology, Santa Cruz, CA, USA). The brain was taken from a mouse that was scarified (with efforts to minimize suffering) for a previous study according to a protocol and recommendations approved by the Committee on the Ethics of Animal Experiments at Rabin Medical Center [24]. Unfortunately, there are no commercially available positive controls for VPAC1-R and VPAC2-R.

Primary antibodies were goat polyclonal antibodies against VIP, VPAC1-R and VPAC2-R, which are reported to be suitable for IMH according to the manufacturer, (Santa Cruz Biotechnology, catalogue numbers: sc-21041, sc-31633 and sc-15961, respectively). The samples were incubated with the primary antibodies diluted 1/10 and 1/30. Negative control solutions were prepared for VIP, VPAC1-R and VPAC2-R by absorption of their primary antibodies with their corresponding blocking peptide (Santa Cruz Biotechnology, catalogue numbers: sc-21041P, sc-31633P and sc-15961P, respectively). Red–brown 3-amino-9-ethylcarbazole (Zymed Laboratories Inc., San Francisco, CA, USA) staining indicated the presence of antigen, with blue Mayer’s hematoxylin (Pioneer Research Chemicals Ltd., Colchester Essex, UK) counterstaining.

### Follicular Counts

The number of follicles in every IMH stained section was counted with an image analyzer (analySIS, Soft Imaging System, Digital Solutions for Imaging and Microscopy, System GmbH, Munster, Germany) [8], [10], [11]. The follicles were classified as follows [25]: *primordial* (with a single flat layer of GC surrounding the oocyte), *primary* (with a single cuboidal GC layer surrounding the oocyte), *secondary* (with at least two GC layers and a theca layer surrounding the oocyte); and antral (with a fluid-filled cavity within the GC). Follicles preceding the antral stage (primordial, primary and secondary) were collectively termed *preantral*.

### RT-PCR

A TRIzol Reagent protocol (Pioneer Research Chemicals) was used for mRNA extraction from frozen ovarian samples, as described in detail elsewhere [8, 10, 11). The concentration of each sample was measured using a spectrophotometer (Cary UV 100; Varian, Mulgrave, Australia), and samples were stored at −80°C until RT-PCR was performed.

Frozen total RNA samples from eight adult and eight fetal ovaries were centrifuged at 13,000 *g* for 30 min at 4°C. After the supernatant was completely removed, the pellets containing RNA were resuspended in 50 µl RNase-free water. The concentration of each sample was measured by spectrophotometer (NanoDrop 2000, Thermo Scientific, Wilmington, DF, USA). The samples were aliquoted, 0.5 µg RNA per PCR tube, and stored at –80°C. A total of 0.5 µg RNA was used for cDNA synthesis in the presence (RT+) or absence (RT–) of reverse transcriptase (Moloney murine leukemia virus, M-MLV RT) according to the manufacturer’s instructions (Invitrogen, catalogue number 280250, Life Technologies, Grand Isler, NY, USA).

The cDNA amplification primers for VIP, VPAC1-R, and VPAC2-R were designed to span at least one intron so as to identify genomic DNA contamination. A single-round PCR approach was used to detect transcripts of VIP (accession number NM_194435.1), VPAC1-R (accession number NM_004624.3), and VPAC2-R (accession number NM_003382.4). To detect VIP, the forward primer (5′-tcaaacgtcactcagatgcag-3′) and reverse primer (5′-tcttctggaaagtcgggaga-3′) were designed in exon 4 and exon 5. The expected PCR product size was 134 base pairs (bp). To detect VPAC1-R, the forward primer (5′-ccggacaattttaagcctga-3′) and reverse primer (5′-accattgaggaagcagtagagg-3′) were designed in exon 11 and exon 12. The expected PCR product size was 97 bp. To detect VPAC2-R, the forward primer (5′-tgtactgagactggtcgttgc-3′) and reverse primer (5′-tgggatacaaacgaccacag-3′) were designed in exon 4 and exon 5. The expected PCR product size was 155 bp. DHFR (accession number NM_000791.3) gene served as a positive control for the RT-PCR. The forward primer (5′-ggttgtggtcattctctggaa-3′) and reverse primer (5′-ctcgcctgcacaaatgg-3′) were designed in exon 5 and exon 6. The expected PCR product size was 231 bp. The annealing temperature for all four PCR reactions was 53°C and for all four the cycle number was 40.

The total 20 µl PCR reaction contained 2 µl of reverse-transcribed cDNA, 10 µl of 2× Master Mix (Fast Cycling PCR Kit, QIAGEN, Ontario, Canada), and 0.2 µM of each primer. PCR was carried out according to the manufacturer’s instruction (QIAGEN, catalogue number 203741). The amplified product was subjected to 4% agarose gel electrophoresis, using a 100 base-pair ladder as a reference (100 bp Ladder, QIAGEN) for fragment size, and stained with ethidium bromide.
